# *In silico* simulations of erythrocyte aquaporins with quantitative *in vitro* validation†

**DOI:** 10.1039/d0ra03456h

**Published:** 2020-06-04

**Authors:** Ruth Chan, Michael Falato, Huiyun Liang, Liao Y. Chen

**Affiliations:** Department of Physics, The University of Texas at San Antonio San Antonio Texas 78249 USA Liao.Chen@utsa.edu; Department of Pharmacology, The University of Texas Health Science Center at San Antonio San Antonio Texas 78229 USA

## Abstract

Modelling water and membrane lipids is an essential element in the computational research of biophysical/biochemical processes such as water transport across the cell membrane. In this study, we examined the accuracies of two popular water models, TIP3P and TIP4P, in the molecular dynamics simulations of erythrocyte aquaporins (AQP1 and AQP3). We modelled the erythrocyte membrane as an asymmetric lipid bilayer with appropriate lipid compositions of its inner and outer leaflet, in comparison with a symmetric lipid bilayer of a single lipid type. We computed the AQP1/3 permeabilities with the transition state theory with full correction for recrossing events. We also conducted cell swelling assays for water transport across the erythrocyte membrane. The experimental results agree with the TIP3P water–erythrocyte membrane model, in confirmation of the expected accuracy of the erythrocyte membrane model, the TIP3P water model, and the CHARMM parameters for water–protein interactions.

## Abstract 2

Quantitatively predictive study of aquaporins in model erythrocyte membrane validated with cellular experiments.

## Graphical abstract

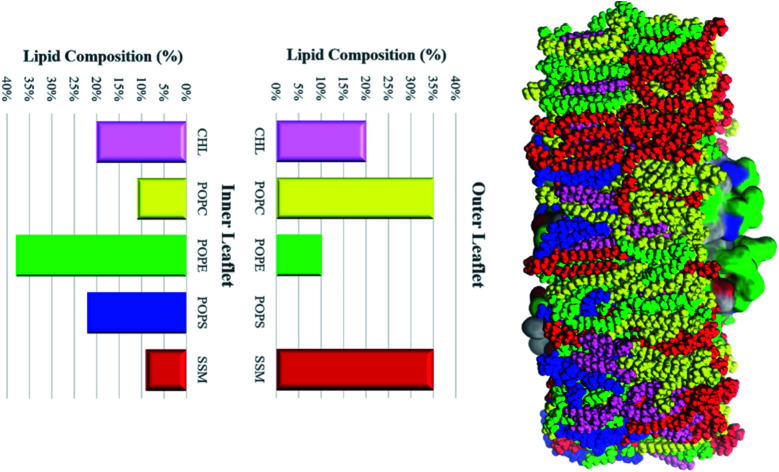

## Introduction

Transport of water across the cellular membrane is an essential biological process, which is facilitated by the water channel proteins, aquaporins (AQPs).^1–8^ Naturally, the literature on AQPs is extremely rich and the research is currently active. See, *e.g.*, ref. 9–31 for some recent studies. Alongside experimental investigations, molecular dynamics (MD) simulations have been carried out to elucidate the molecular/atomistic insights that cannot be gained from experiments alone.^32–39^ Now the copy numbers of the AQPs natively expressed in the human erythrocyte are known quantitatively,^40^ can we make quantitatively accurate predictions of their transport characteristics from the all-atom MD simulations? Is it necessary to model the erythrocyte membrane with appropriate lipid compositions in its inner and outer leaflets^41–46^? In another word, what difference would it be if the membrane is simply modelled as a lipid bilayer of a single lipid type as it is in the current literature? Relatedly, which simple water model, TIP3P^47,48^ or TIP4P/2005,^49,50^ gives a more accurate representation of the essential solvent, water, in the study of water channels? In this article, we answer these questions with the following *in silico* and *in vitro* investigations. Our *in silico*–*in vitro* studies show that it is necessary to model the AQPs in an erythrocyte membrane with the experimentally validated lipid compositions^41–46^ of the inner and the outer leaflets and that the all-atom model with TIP3P is quantitatively accurate.

We conducted all-atom MD simulations of AQP1 and AQP3 that are natively expressed in the human erythrocyte. Each model system consists of a biologically functional unit of the channel proteins (AQP1/3 tetramer) embedded in a patch of erythrocyte (noted as RBC hereafter) membrane with the lipid composition tabulated in Table 1, which have been experimentally validated and used in molecular studies in the current literature.^41–46^ We used both the TIP3P water model and the TIP4P/2005 water model in respective studies. TIP3P was found to be more accurate for water transport through AQPs than TIP4P/2005 even though the latter gives better estimate for the bulk water properties. This is understandable because water conduction through AQPs is largely determined by the protein–water interactions represented by the CHARMM force parameters that are optimized with the TIP3P water model. For comparison, we also conducted simulations of AQP1 and AQP3 embedded in a patch of phosphatidylethanolamine (POPE) bilayer using TIP3P. In quantitative differentiations of the all-atom MD simulations, we conducted *in vitro* experiments on human erythrocytes. Using light scattering off the erythrocyte mixtures in a stopped-flow device, we quantified the cellular transport process with known accuracy. The experimental data validate our *in silico* study of erythrocyte membrane model with TIP3P and CHARMM36 parameters. The experiments further illustrate that AQPs are not modulated by the erythrocyte conformational changes. From this *in silico*–*in vitro* investigation of erythrocyte aquaporins, we observe that theoretical–computational research can produce quantitatively accurate predictions for the transport of water and solutes across the cell membrane. Given today's high-performance supercomputing power, it is feasible to atomistically model the cell membrane in close approximation of the reality. Accurate and new insights can be gained by learning from big-data sets of experimental assays beyond the conventional ways of oversimplifying them in experimental analyses.

**Table 1:** Lipid compositions of the model erythrocyte membrane (RBC)

	Cholesterol (CHL)	Phosphatidyl-choline (POPC)	Phosphatidyl-ethanolamine (POPE)	Phosphatidyl-serine (POPS)	Sphingomyelin (SSM)
Inner leaflet	20%	11%	38%	22%	9%
Outer leaflet	20%	35%	10%	0%	35%

## Experimental methods

### System setup and simulation parameters

**Fig. 1:**
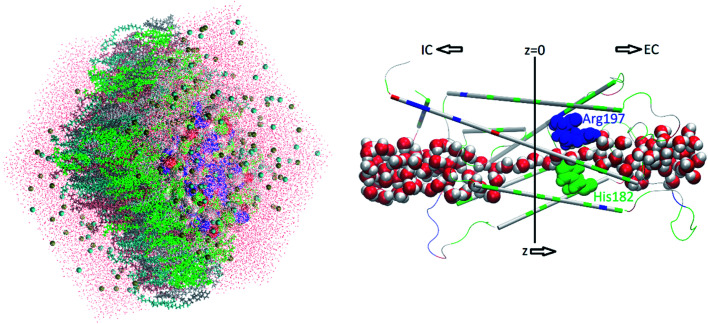
All-atom model system of AQP1 tetramer in erythrocyte membrane solvated with TIP3P waters. The full system is shown in the left panel where waters are in lines colored by atoms (O, red; H, white); NaCl ions are shown in spheres colored by atoms; protein in surface colored by residue types; and lipids in licorices colored by residue names. In the right panel, one monomer channel protein is shown in thin cartoons with the aromatic/arginine selectivity filter (ar/R sf) residues (Arg 197 and His 182) in large spheres, all colored by residue types. Water molecules inside and near the channel are shown in large spheres colored by atoms. The *z*-axis points from the intracellular (IC) side (*z* <−15 Å) to the extracellular (EC) side (*z* > 15 Å). All the molecular graphics of this article were rendered with VMD.^57^

Following the well-tested steps of the literature, we employed CHARMM-GUI^51^ to set up six all-atom model systems tabulated in Table 2. In each system, the AQP1/3 tetramer was embedded in a patch of membrane. The AQP1 coordinates were taken from PDB: 1J4N^52^ (crystal structure) and the AQP3 coordinates were taken from ref. 29 (optimized homology model validated with *in vitro* experiments). The positioning of the AQP tetramer was determined by matching the hydrophobic side surface with the lipid tails and aligning the channel axes perpendicular to the membrane. The AQP-membrane complex was sandwiched by two layers of TIP3P/TIP4P waters, each of which is approximately 35 Å thick. The system was then neutralized and salinated with Na^+^ and Cl^−^ ions to a salt concentration of 150 mM. A representative system is illustrated in Fig. 1. We employed NAMD 2.13 (ref. 53) as the MD engine. We used CHARMM36 parameters^54–56^ and the TIP3P or TIP4P/2005 parameters for inter- and intra-molecular interactions. After the initial equilibration steps, we conducted unbiased MD production runs (150 ns for each system) with constant pressure at 1.0 bar (Nose–Hoover barostat) and constant temperature at 298.15 K (Langevin thermostat). The Langevin damping coefficient was chosen to be 1 ps^−1^. The periodic boundary conditions were applied to all three dimensions. The particle mesh Ewald was used for the long-range electrostatic interactions (grid level: 128 × 128 × 128). The time step was 2.0 fs. The cut-off for long-range interactions was set to 10 Å with a switching distance of 9 Å. The last 100 ns of the trajectories were used in the computation of transport characteristics. (All the coordinates, parameters, and scripts necessary to reproduce the results of this study is available at https://www.doi.org/10.7910/DVN/YGO6HG.)

**Table 2:** Simulation systems

Model system	Number of atoms	System dimensions	Simulation times
AQP1–TIP3P–RBC membrane	168 365	117 Å × 117 Å × 121 Å	50 ns + 100 ns
AQP1–TIP3P–POPE bilayer	134 876	107 Å × 107 Å × 119 Å	50 ns + 100 ns
AQP1–TIP4P–RBC membrane	203 801	116 Å × 118 Å × 118 Å	50 ns + 100 ns
AQP3–TIP3P–RBC membrane	158 896	118 Å × 118 Å × 112 Å	50 ns + 100 ns
AQP3–TIP3P–POPE bilayer	153 080	111 Å × 111 Å × 123 Å	50 ns + 100 ns
AQP3–TIP4P–RBC membrane	190 884	112 Å × 112 Å × 120 Å	50 ns + 100 ns

It is interesting to note that water molecules line up in single file inside an AQP channel (illustrated in Fig. 1, right panel). AQP1 has these water molecules in the crystal structure, which were kept in the AQP1 model systems. The AQP3 model systems do not have these water molecules in the initial frame of coordinates. However, it took about 1.0 ns MD run for the AQP3 channels to become filled with water molecules. All transient effects from the initial coordinates were excluded from our computation by discarding the first 50 ns of the trajectories of a 150 ns MD run for each system (Table 2).

### Transition state theory for water permeability

It has been clearly illustrated in the literature that an imbalance of osmolyte concentrations between the two sides of a membrane generates a difference/gradient in the chemical potential of water molecules. It is such a chemical potential gradient that drives the osmotic flux of water across the membrane (through AQPs) that is impermeable to the osmolyte solutes. Specifically, consider the extracellular (EC) and the intracellular (IC) sides of an erythrocyte and use *c*_e_ and *c*_i_, respectively, to denote the EC and the IC concentrations of the impermeable solutes. The difference in water chemical potential between the two sides,^33^*μ*_e_ − *μ*_i_ = (*p*_e_ − *p*_i_)*v*_W_ − *RT*(*c*_e_ − *c*_i_)*v*_W_. Here *p*_e_ − *p*_i_ is the difference in mechanic/hydraulic pressure between the two sides; *R* is the gas constant; *T* is the absolute temperature; and *v*_W_ is the molar volume of water. The IC-to-EC transition rate constant of water *k*_I to E_ is related to the EC-to-IC rate constant *k*_E to I_: *k*_I to E_ = *k*_E to I_ exp[−(*μ*_e_ − *μ*_i_)/*RT*]. In the absence of a hydraulic pressure difference, *p*_e_ − *p*_i_ = 0, *k*_I to E_ = *k*_E to I_ exp[(*c*_e_ − *c*_i_)*v*_W_]. Under a hyperosmotic condition, *c*_e_ − *c*_i_ > 0, *k*_I to E_ > *k*_E to I_. In this case, the outward chemical potential gradient induces a net efflux of water. The corresponding transition rate (transitions per unit time facilitated by one AQP channel) *r* = *k*_I to E_(1/*v*_W_ − *c*_i_) − *k*_E to I_(1/*v*_W_ − *c*_e_). Note that 1/*v*_W_ is the concentration of water molecules in the absence of solutes. The presence of solutes reduces the water concentration. Furthermore, the validity of these formulas is limited to the dilute solution regime, namely, (*c*_e_ − *c*_i_)*v*_W_ ≪ 1, which is quantitatively accurate for osmolyte concentration in the sub Molar range. In this regime, the linear expansion in terms of (*c*_e_ − *c*_i_) is valid, which leads to the transition rate *r* = 2*k*_0_(*c*_e_ − *c*_i_). Therefore, the water flux through a single AQP channel (the volume of water flowing through a channel per unit time), *J* = *rv*_W_/*N*_A_ = 2*k*_0_(*c*_e_ − *c*_i_)*v*_W_/*N*_A_. Correspondingly, the single-channel permeability *p*_f_ = *J*/(*c*_e_ − *c*_i_) = 2*k*_0_*v*_W_/*N*_A_. Here *k*_0_ is the rate constant *k*_0_ = *k*_E to I_ = *k*_I to E_ at equilibrium (*c*_e_ = *c*_i_) and *N*_A_ is the Avogadro number.

However, the rate constant of IC-to-EC or EC-to-IC transition cannot be simply computed as the rate of water molecules crossing the dividing plane between the IC and the EC sides (*z* = 0, Fig. 1) because the potential energy landscape of the water–protein-membrane system is very rugged^58^ and thus there are many recrossing events that need to be considered^59,60^ in the application of the transition state theory (TST) to compute the transition rate constant.^61^ Many of the events of a water molecule crossing the dividing plane actually end up recrossing the plane in the opposite direction and thus should be excluded from the counting of transitions between the IC and the EC sides. The IC-to-EC transitions consist of only the events of crossing the dividing plane in the positive *z*-direction and continuing onto the EC side without recrossing the dividing plane. Likewise, the EC-to-IC transitions. Therefore, TST gives *k*_0_ = *κ* < *v*_*z*_*δ*(*z* − *z*_0_) > |_*v*_*z*_>0_ where *κ* is the correction factor and *z* = *z*_0_ = 0 (Fig. 1) is the dividing plane. The brackets indicate equilibrium statistical average of the velocity along the *z*-direction (or, equivalently, the negative *z*-direction for the EC-to-IC rate constant. Noting again that *k*_0_ = *k*_E to I_ = *k*_I to E_ at equilibrium). Carrying out the equilibrium statistical average, we have 
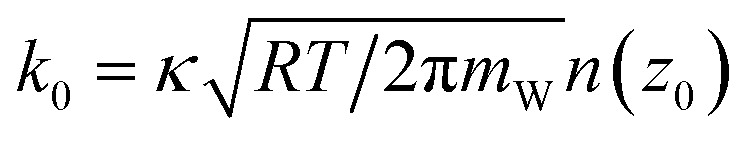
 with *m*_W_ being the molar mass of water. The linear density of water at the dividing plane *n*(*z*_0_) can be readily evaluated with equilibrium sampling. The evaluation of the correction factor *κ* is computationally expensive. *κ* = 1 when the dividing plane *z* = *z*_0_ is the perfect transition state for a water molecule. If we had such a perfect transition state, once a molecule crosses the dividing plane along the *z*-direction, it will go on to the EC bulk. *Vice versa*, once a molecule crosses the dividing plane along the opposite direction, it will go on to the IC bulk. In our study of water transport through an AQP channel, there is practically not a perfect transition state and, as such, we did not try to locate it. Instead, we evaluated the correction factor *κ* in the following manner: for each event of a molecule crossing the dividing plane from the IC side (*z* < 0) to the EC side (*z* > 0), we followed its trajectory until it either recrosses the plane back to the IC side or moves out of the channel into the EC bulk (*z* > 15). For each event of a molecule crossing the plane along the opposite direction, we followed its trajectory until it either recrosses the plane back to the EC side or goes on to the IC bulk (*z* <−15). The correction factor *κ* is equal to the number of successful transport events (ending up in the EC/IC bulk) divided by the total number of events of crossing the dividing plane. We reached convergence of the statistics for the correction factor and the linear density. In this way, we computed the single-channel permeability of an AQP with known margin of error. It is gratifying to note that the final result of permeability so obtained is insensitive to the exact location of the dividing plane as long as *z*_0_ is inside the channel.

### Experimental procedure and analysis

The packed red blood cells from three anonymous healthy donors were purchased from the South Texas Blood and Tissue Center. Equal volumes of the three erythrocyte samples were mixed and washed three times and then suspended in phosphate buffered saline (PBS) to a total concentration of 4% hematocrit. In each experimental run, 5 μL of the 4% hematocrit suspension in PBS was rapidly mixed with an equal volume of PBS containing 180 mM sucrose using an Applied PhotoPhysics SX20 stopped-flow spectrometer. In such a mixture solution, the extracellular environment of the erythrocyte is hyperosmotic with a gradient of 90 mM which drives a water flux out of the cell resulting in cellular shrinkage. The intensity of light scattered at 90° was measured to monitor the erythrocyte shrinking process. The scattered light intensity is related to the varying cellular volume as follows:*I*(*t*) = 1 + *b*(*V*_0_/*V*(*t*) − 1).Here *t* is time. *b* is a parameter to account for the number of erythrocytes in the samples of a given set of experiments. *V*(*t*) is the intracellular volume of an erythrocyte with its initial value noted as *V*_0_. It follows the following dynamics equation:d*V*/d*t* = *P*_f_*Av*_W_(*c*_i_0__*V*_0_/*V* − *c*_e_).Here *P*_f_ is the osmotic permeability of the entire cell and *A* is the cellular surface area. *c*_i_0__ is the initial intracellular concentration of impermeable solutes.

In an exponential fitting, we followed what is customary in the literature and assumed that the scattered light intensity to be an exponential function of time *I*(*t*) = *A* + *B* exp[−*k*_fit_*t*] that has three fitting parameters *A*, *B*, and *k*_fit_. We conducted least squares fit to obtain the rate *k*_fit_ which is assumed to be proportional to the cellular permeability. It has been pointed out in the literature that the exponential fitting for cellular permeability can be problematic.^62^

In contrast to the exponential fit, we numerically integrated the differential equation of the full dynamics for the time course of scattered light intensity *I*(*t*) for each set of values for the two fitting parameters *b* and *P*_f_*A*. All the other quantities involved (the water molar volume, the initial volume of the erythrocyte, the intracellular and extracellular concentrations of the impermeable solutes) were either known or measured at the time of experiment. We conducted least squares fit to obtain the mean and the standard error of the total cellular permeability *P*_f_*A* without any other assumptions.

## Results and discussion

### Bulk water diffusion

To confirm the literature on the bulk water diffusion constants, we conducted 100 ns MD simulations for a 100 Å × 100 Å × 100 Å box of water represented as TIP3P^47,48^ and TIP4P/2005 (ref. 49 and 50) models respectively. The diffusion constant computed from the mean squared displacements of a water molecule (shown in ESI, Fig. S1†) are 5.8 × 10^−5^ cm^2^ s^−1^ for TIP3P and 2.2 × 10^−5^ cm^2^ s^−1^ for TIP4P/2005, respectively. Indeed, the TIP3P estimate exaggerates the bulk water diffusion by a factor of 2.5 while the TIP4P/2005 estimate is nearly identical to the experimental data (2.3 × 10^−5^ cm^2^ s^−1^). This substantial difference between the two water models naturally leads to the question about the validity of the computation of water conduction through AQPs using the CHARMM force field parameters including the TIP3P parameters. Would TIP4P/2005 give a quantitatively more accurate estimate? The answer should be yes if the water conduction through AQPs is mostly determined by water–water interactions rather than water–protein interactions.

### AQP1/3 permeabilities

**Fig. 2:**
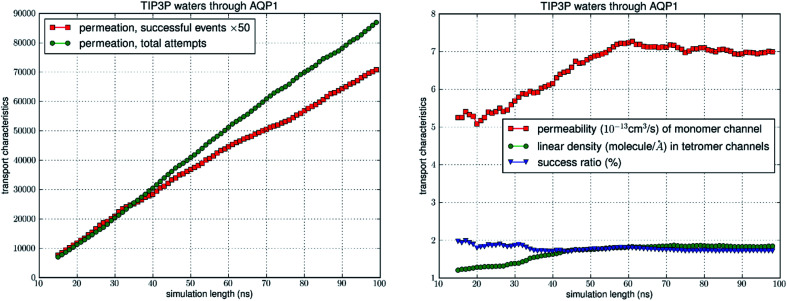
Transport characteristics of erythrocyte AQP1. The left panel shows the time course of the number of transport events *vs.* the number of attempts. The right panel shows convergence of the computation: the computed values of permeability, linear density, and success ratio *vs.* length of simulation.

In equilibrium, water molecules fill the AQP channels in approximately single files (illustrated in Fig. 1). They constantly fluctuate back and forth inside the channel (on the picosecond time scale) and move out of and into the channel (on the sub-nanosecond time scale). The water flux across the dividing plane (*z* = 0) fluctuates between the positive *z*- and the negative *z*-directions to give rise to zero net flux in the absence of an osmotic gradient across the cellular membrane. The single channel permeability *p*_f_ quantifies how readily water molecules can move across the membrane through a water channel in response to an osmotic imbalance between the IC and the EC sides. It is equal to the molar volume of water multiplied by the rate of IC-to-EC transition (or, equally, the rate of EC-to-IC transition) given by the TST formula with the correction for recrossing events. We implemented this correction for recrossing events and found the success ratio to be less than 2% for all six sets of simulations (four AQP1/3-RBC–TIP3/4P combinations and two AQP1/3-POPE–TIP3P combinations). The computational details are illustrated in Fig. 2 for the AQP1–RBC–TIP3P model system and in ESI, Fig. S2 and S3† for the other five model systems. The computed values of single channel permeability at 25 °C are tabulated in Table 3. Two of the six sets are respectively for AQP1 and AQP3 embedded in the model erythrocyte membrane with TIP3P, and similarly another two with the TIP4P/2005 water model. The remaining two sets are respectively for AQP1 and AQP3 embedded in the POPE bilayer with TIP3P. It is interesting to note that the permeability of aquaglyceroporin AQP3 (that conducts glycerol and water) is higher than AQP1 that is water specific and does not conduct glycerol.

**Table 3:** Computed AQP1/3 permeabilities *vs.* experimental data on erythrocytes

	In POPE-bilayer, computed with TIP3P	In RBC membrane, computed with TIP3P	In RBC membrane, computed with TIP4P
*p* ^AQ1^_f_ per AQP1 monomer	3.5 × 10^−13^ cm^3^ s^−1^	7.0 × 10^−13^ cm^3^ s^−1^	2.4 × 10^−13^ cm^3^ s^−1^
*p* ^AQ3^_f_ per AQP3 monomer	3.7 × 10^−13^ cm^3^ s^−1^	8.1 × 10^−13^ cm^3^ s^−1^	4.4 × 10^−13^ cm^3^ s^−1^
Computed RBC permeability^a^	(2.1 ± 0.5^b^) × 10^−8^ cm^3^ s^−1^	(4.2 ± 0.7^b^) × 10^−8^ cm^3^ s^−1^	(1.7 ± 0.4^b^) × 10^−8^ cm^3^ s^−1^
Experimentally measured RBC permeability (*P*_f_*A*)^c^	(4.3 ± 0.2) × 10^−8^ cm^3^ s^−1^		

a *P*_f_ *A* = (*N*^AQ1^*p*^AQ1^_f_ + *N*^AQ3^*p*^AQ3^_f_) with the numbers of AQP1/3 copies per erythrocyte, *N*^AQ1^ = (58 ± 10) × 10^3^ and *N*^AQ3^ = (2.0 ± 0.5) × 10^3^, which were taken from ref. 40.

b The error margins largely reflect those from the proteomics study of ref. 40 because the simulations gave much smaller error bars.

c This experimental value represents the mean between two sets of experiments shown in Fig. 3 (on erythrocytes in 1 × PBS) and Fig. 4, top panel (on erythrocytes in 0.7 × PBS), respectively.

For AQP1/3 in the model erythrocyte membrane, the use of TIP3P and the use of TIP4P/2005 produced results that differ by a factor of about two. These differences are outside the margins of error of our computation and also outside the margin of error of our experimental data. Considering the model erythrocyte membrane *vs.* the lipid bilayer of a single lipid type, the results for AQP1 and AQP3 are also significantly different, outside the margins of error. Therefore, our experiments can be applied to quantitatively validate the AQP1/3-RBC-membrane-TIP3P model.

### Experimental data

To differentiate these *in silico* models, we conducted *in vitro* experiments of erythrocyte shrinking in a hyperosmotic medium probed with the stopped-flow light scattering spectrometer. A small number of (but representative) data points are shown in Fig. 3. From the big data (10 000 data points in each of the 20 or more experimental runs conducted for each condition), we learnt that the process of erythrocyte shrinking in response to hyperosmotic mixing does not precisely follow the exponential behavior (red curve in Fig. 3) that was generally assumed in the current literature. Instead, we can learn the time-course (black curve in Fig. 3) from the data with minimal assumptions that are certainly invariable: the number of AQPs in a given erythrocyte did not change during the shrinkage process and the simple laws of hydrodynamics cannot be altered while the cellular shape and volume are unknown variables. Using the total permeability of a cell *P*_f_*A* as the fitting parameter, we obtained the full–dynamics curve along with the mean value and the standard error of *P*_f_*A* shown in Fig. 3 and Table 3. With this, we can conclude that the *in silico* model system of AQP1/3-RBC–TIP3P is validated by the *in vitro* experiments on human erythrocytes. The other model systems (AQP1/3-POPE–TIP3P or AQP1/3-RBC–TIP4P) deviate from the experimental data by a factor between two and three. At this point, one may ask: does the erythrocyte surface tension play a significant role in the cellular shrinking process? The surface tension can cause a mechanic pressure across the erythrocyte membrane which amounts to less than 0.72 kPa. This can be safely neglected in our study because the osmotic pressure induced by 90 mM sucrose is approximately 220 kPa.

**Fig. 3:**
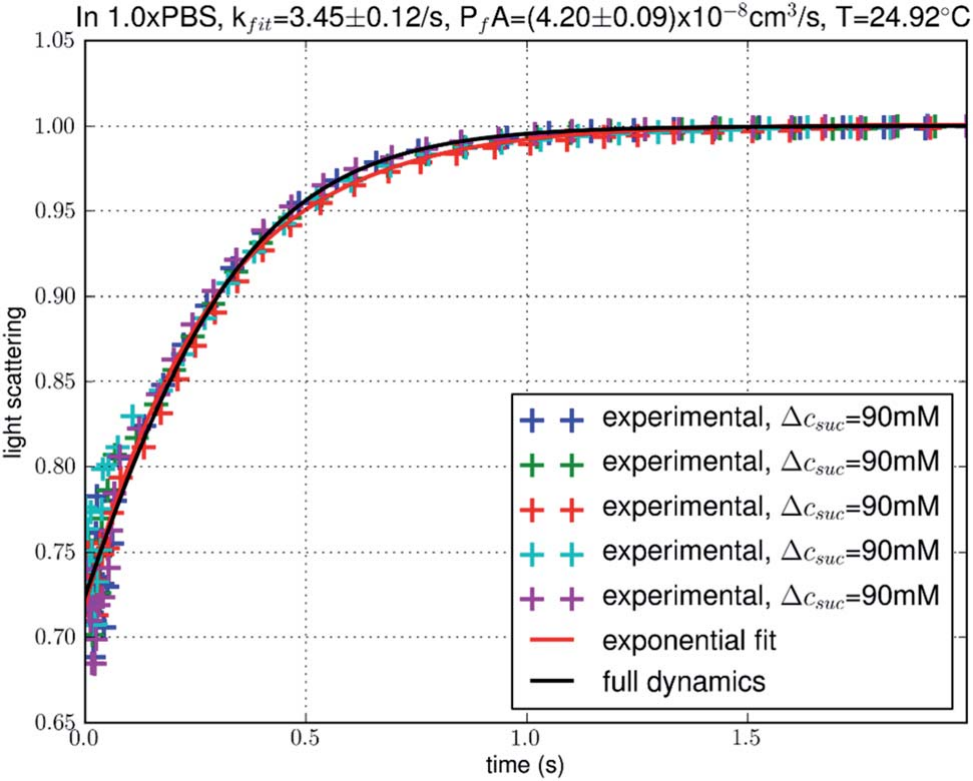
Experimental data of the time courses of human erythrocyte shrinking. The solid curves represent the theoretical predictions from the simple rate equation of osmotic water flux caused by an outward/inward 90 mosM gradient with total water flux *P*_f_*A* as a fitting parameter. The crosses represent normalized intensity of 90° scattered light. Light intensity is normalized with the final constant level in each experiment.

**Fig. 4:**
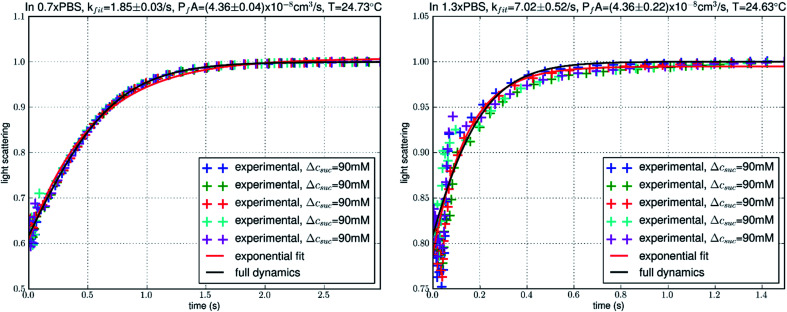
Additional experiments. All conditions are identical to Fig. 3 except that the buffers used were 0.7 × PBS (left) and 1.3 × PBS (right) respectively.

**Fig. 5:**
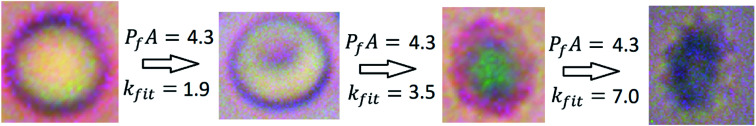
Invariance of AQP permeability illustrated. The numbers indicate total membrane permeability *P*_f_*A* = 4.3 × 10^−8^ cm^3^ s^−1^ and the exponential fitting rate constant *k*_fit_ = 1.9 s^−1^, 3.5 s^−1^, or 7.0 s^−1^ respectively. The optical images were taken of RBC in 270 mosM (left image), 300 mosM (second from left image), 390 mosM (third from left image), and 480 mosM (right image) saline-sucrose solutions respectively (images not beautified with optical filtering).

It is interesting to examine whether the membrane permeability of erythrocytes depends on the cell volume and conformation. For that purpose, we repeated the erythrocyte shrinking experiments with 0.7 × PBS and 1.3 × PBS as the buffer liquids respectively. The results shown in Fig. 4, in comparison with Fig. 3, demonstrated that *P*_f_*A* is invariant among the three sets of experiments. Considering that the cellular volume and conformation varied significantly from one set of experiments to another, we conclude that water transport across the erythrocyte membrane is, within the margin of error, all through aquaporins. Otherwise, the value of *P*_f_*A* would vary with the membrane surface area. We further concluded that aquaporins are not modulated by the cell volume-conformation (illustrated in Fig. 5).

### Quantitative significance of lipid compositions

**Fig. 6:**
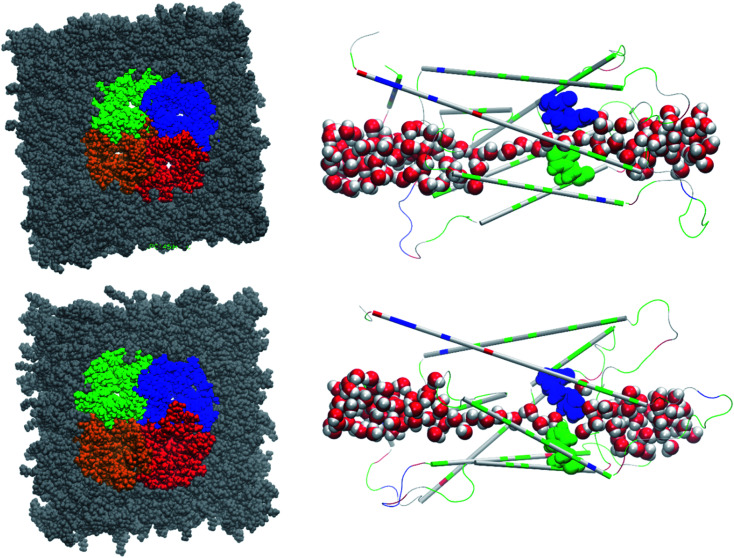
Water conduction through AQP1 in the erythrocyte membrane (top two panels) *vs.* that in POPE bilayer (bottom two panels). In the right two panels, the protein monomer is shown in thin cartoons with the ar/R sf residues (Arg 197 and His 182) in large spheres, all colored by residue types (hydrophilic, green; hydrophobic, white; negatively charged, red; positively charged, blue). Water molecules inside and near the channel are shown in large spheres colored by atoms (O, red; H, white). In the left two panels, the protein tetramers (colored by protein monomers) and the membrane lipids (in gray) are all shown in space-filling spheres, viewed from the extracellular side.

**Fig. 7:**
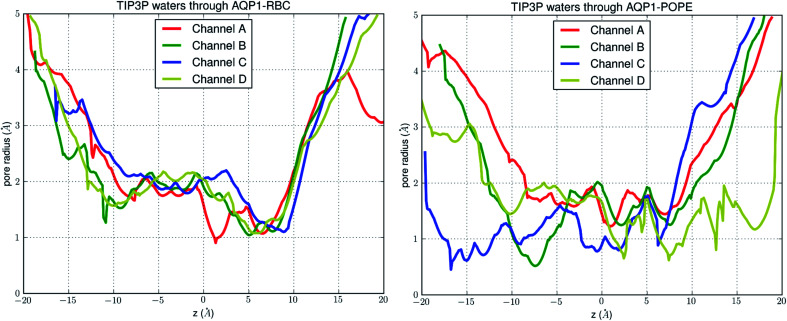
Pore radii of the AQP1 tetramer channels in the erythrocyte membrane (left panel) *vs.* in POPE bilayer (right panel). The coordinates were taken from a snapshot at 120 ns of the 150 ns MD run. At this moment, water transport is interrupted in channel B, C, and D of the AQP1–POPE system. The radii were measured using the HOLE program.^63^

The AQP1/3-RBC–TIP3P simulations produced quantitative agreement with the experimental data while the AQP1/3-POPE–TIP3P simulations underestimated the cellular permeability by a factor of two. Fig. 6 and 7, and Movies 1 and 2† show how that happened. Looking into the stochastic dynamics of the water molecules interacting with the proteins which are surrounded by the membrane lipids, we noticed that, in a typical frame of the trajectory, all four monomer channels of AQP1 in the model erythrocyte membrane are constantly open for water flux while, on the average, two of the four channels of AQP1 in the POPE bilayer are closed. Why? The erythrocyte membrane has a tighter fit for the membrane proteins, causing AQP1 residues at the ends of the channel to have smaller fluctuations. In contrast, the POPE bilayer is loose, which allows greater fluctuations of the AQP1 residues near the channel ends. This can be seen from the left panels of Fig. 6. Quantitatively, the mean square fluctuations of the channel end residues are shown in ESI, Fig. S4.† It is interesting to note that the head group of phosphatidylcholine (POPC) is bulkier than POPE. Expectedly, POPC bilayer should behave somewhere in between POPE bilayer and erythrocyte membrane. The water permeability through AQP1 in POPC bilayer was found to be 4.2 × 10^−13^ cm^3^ s^−1^ (ESI, Fig. S5†) in comparison with 3.5 × 10^−13^ cm^3^ s^−1^ for AQP1 in POPE bilayer and 7.0 × 10^−13^ cm^3^ s^−1^ for AQP1 in erythrocyte membrane. Indeed, the tight fit of AQP1 in the erythrocyte membrane is in fact optimal to give the greatest permeability of the channel protein.

It is also interesting to note that water permeates through an AQP1 channel in single file as illustrated in Fig. 1 and 6 (right panels). The aromatic/arginine (ar/R) residues located on the EC side of the AQP1 channel form the selectivity filter (sf) that allows only water molecules to pass through. The larger fluctuations of the EC end residues of AQP1 in POPE bilayer lead to more fluctuations of the ar/R sf between allowing water passage there at one moment and interrupting water passage there at another moment. When the ar/R sf is in a state of allowing water passage there, the pore radius is >∼1 Å. Otherwise, the pore radius <1 Å. Fig. 7 shows such a moment when all four channels of AQP1–RBC system are conducting water while three of the four channels of AQP1–POPE are not.

## Conclusions

For water transport through erythrocyte aquaporins, quantitative agreement can be reached between the *in vitro* experiments and the *in silico* simulations if the erythrocyte membrane is modelled with appropriate lipid compositions in its inner and outer leaflets. The TIP3P water model is more accurate than TIP4P for the aquaporin simulations even though the latter gives a better estimate of the bulk water properties. Unlike many other membrane proteins, aquaporins are not modulated by the changes in cellular volume and conformation.

## Data availability

The Dataset (parameters, coordinates, scripts, *etc.*) to replicate this study is available at Harvard Dataverse, https://www.doi.org/10.7910/DVN/YGO6HG.

## Author contributions

RC, MF and LYC did the computational work; RC and HL did the experimental measurements; LYC conceptualized the research and wrote the paper; all participated in analyzing the data and editing the manuscript.

## Grant support

This work was supported by the NIH (GM121275).

## Conflicts of interest

There are no conflicts to declare.

## Supplementary Material

RA-010-D0RA03456H-s001

RA-010-D0RA03456H-s002

RA-010-D0RA03456H-s003
