# Supplementary material for: In silico simulations of erythrocyte aquaporins with quantitative in vitro validation
Source: RSC Adv. 2020 Jun 4;10(36):21283–91. doi: 10.1039/d0ra03456h (PMC7328926; doi:10.1039/d0ra03456h)
Supplement: RA-010-D0RA03456H-s003 [file RA-010-D0RA03456H-s003.pdf]

Electronic Supplementary Material (ESI) for RSC Advances.
This journal is © The Royal Society of Chemistry 2020

**Supplemental Materials**

# *in silico* simulations of erythrocyte aquaporins with quantitative *in vitro* validation

Ruth Chan[1], Michael Falato[1], Huiyun Liang[1,2], and Liao Y. Chen[1*]

[1]Department of Physics, The University of Texas at San Antonio, San Antonio, Texas 78249 USA

[2]Department of Pharmacology, The University of Texas Health Science Center at San Antonio, San Antonio, Texas 78229 USA

Here we present additional figures that are referred to but not included in the main text:

In Fig. S1, we show the simulation data of bulk water diffusion with TIP3P vs TIP4P.

In Figs. S2a and b, we show the simulation data of AQP1 embedded in POPE bilayer;

In Figs. S2c and d, we show the simulation data of erythrocyte AQP1 but water modelled with TIP4P;

In Figs. S3a and b, we show the simulation data of erythrocyte AQP3;

In Figs. S3c and d, we show the simulation data of AQP3 embedded in POPE bilayer;

In Figs. S3e and f, we show the simulation data of erythrocyte AQP3 but water modelled with TIP4P;

In Fig. S4, we show fluctuations of the non-helix residues of AQP1 in erythrocyte membrane *vs* in POPE bilayer;

In Figs. S5a and b, we show the simulation data of AQP1 embedded in POPC bilayer.

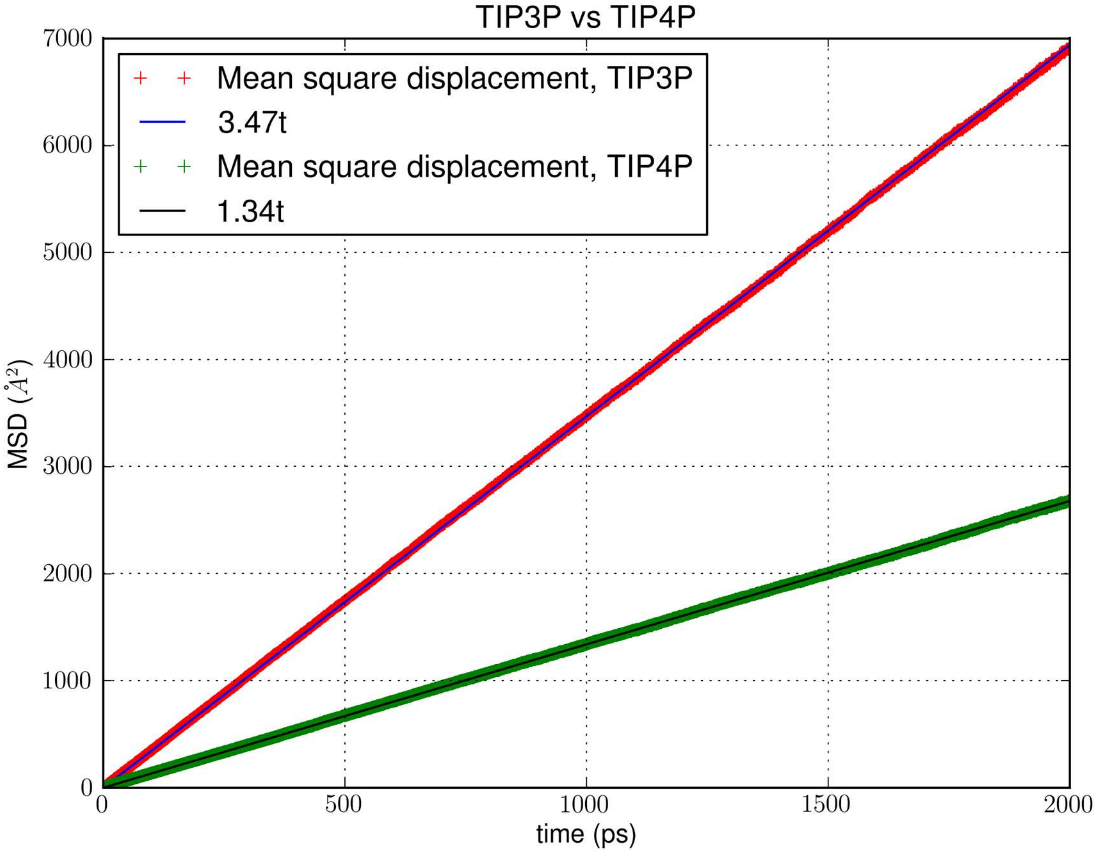


**Fig. S1.** Diffusion in bulk water modelled as TIP3P *vs* TIP4P: Mean squared displacement of a water molecule as a function of the simulation time. For each water model, the simulation was 100 ns in length and was folded into 50 sets of data over 2000 ps.

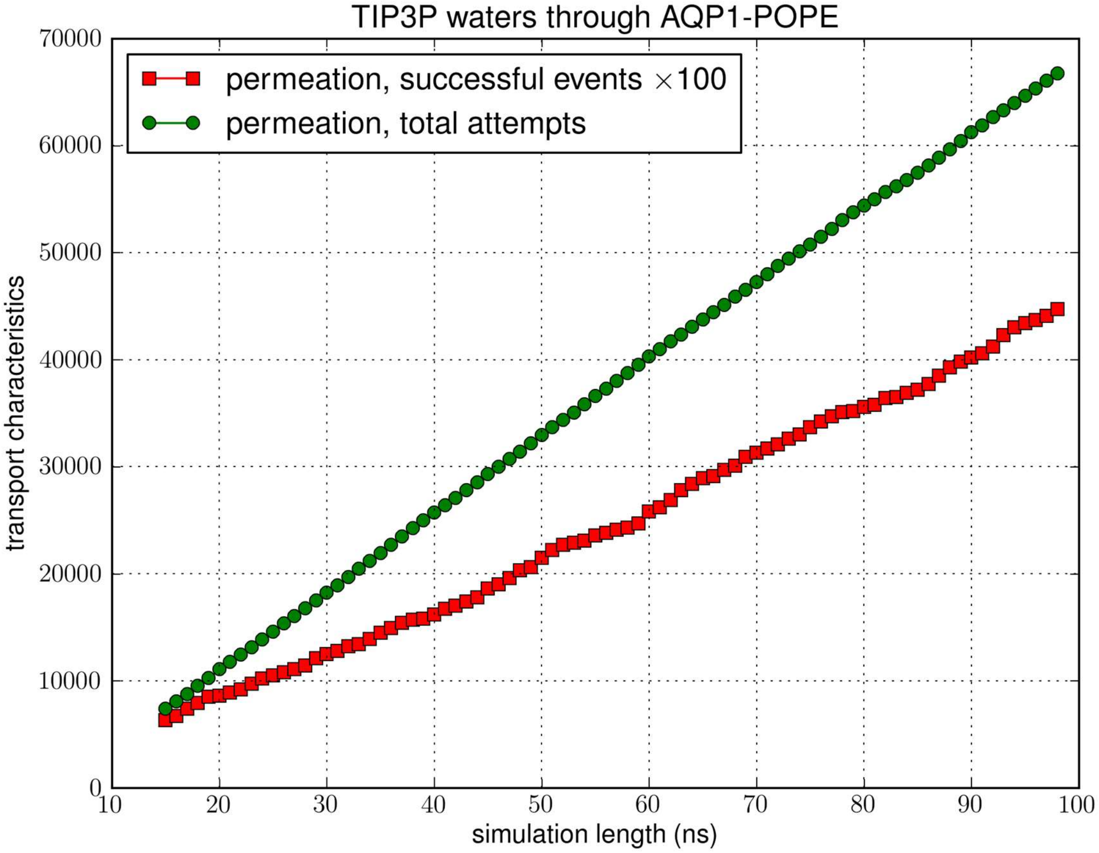


**Fig. S2a**. Transport characteristics of AQP1 embedded in POPE bilayer: the time course of the number of transport events *vs.* the number of attempts.

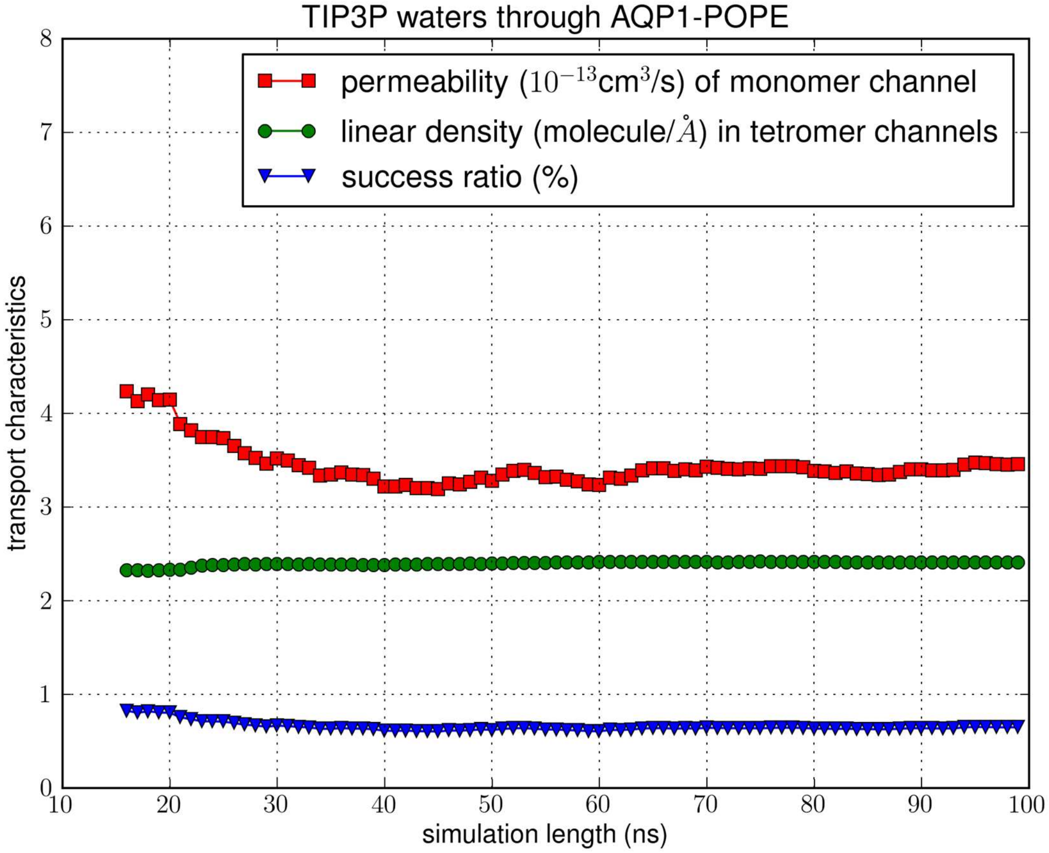


**Fig. S2b**. Transport characteristics of AQP1 embedded in POPE bilayer: convergence of the computation: the computed values of permeability, linear density, and success ratio *vs.* length of simulation.

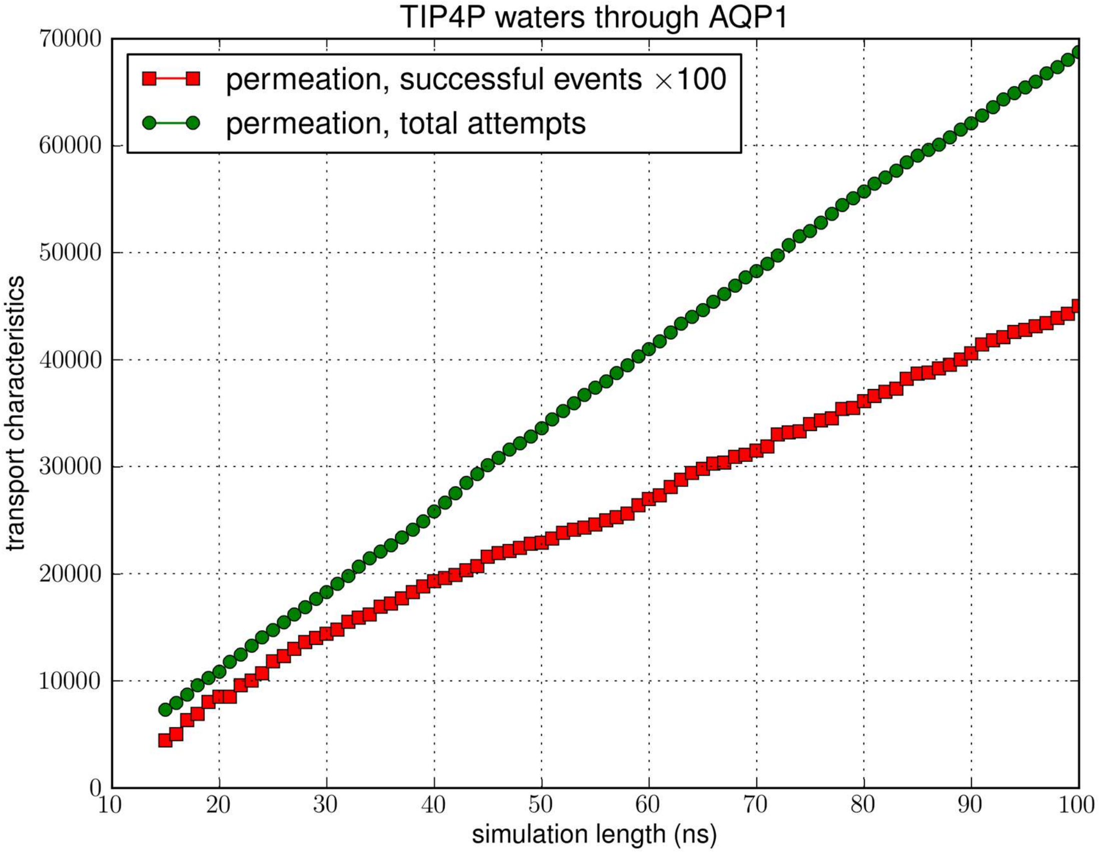


**Fig. S2c**. Transport characteristics of erythrocyte AQP1 with TIP4P water model: the time course of the number of transport events *vs.* the number of attempts.

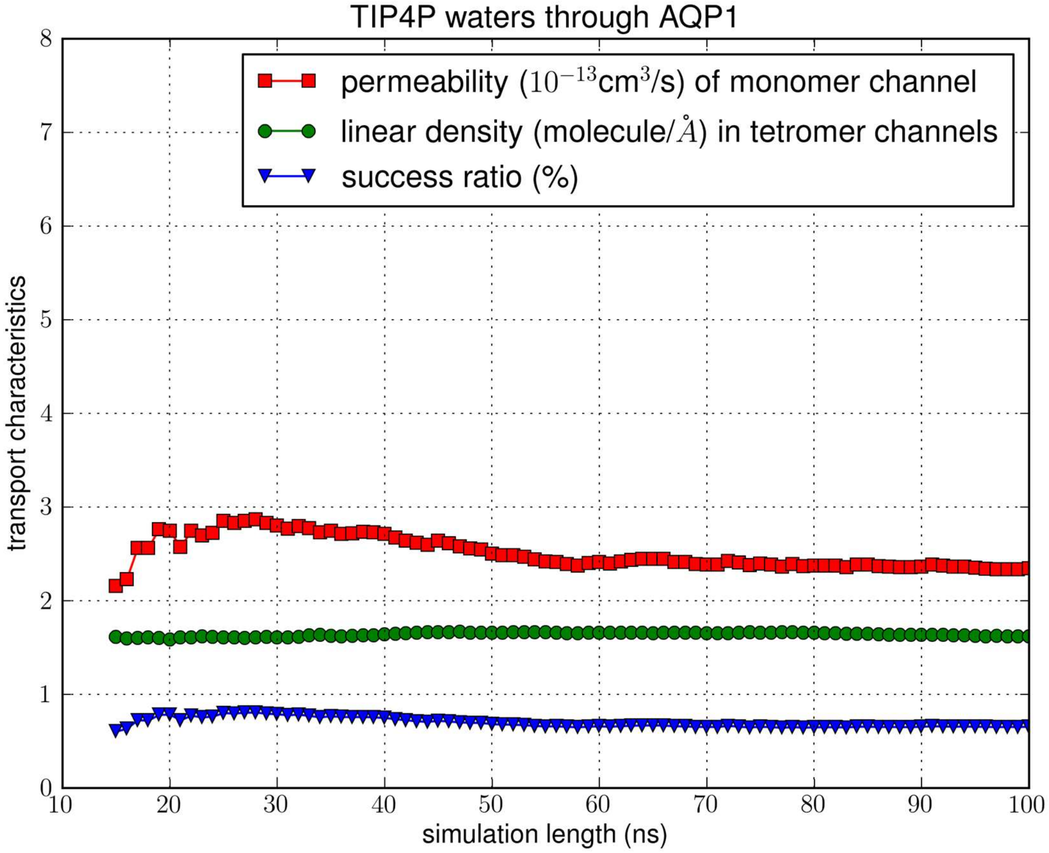


**Fig. S2d**. Transport characteristics of erythrocyte AQP1 with TIP4P water model: convergence of the computation: the computed values of permeability, linear density, and success ratio *vs.* length of simulation.

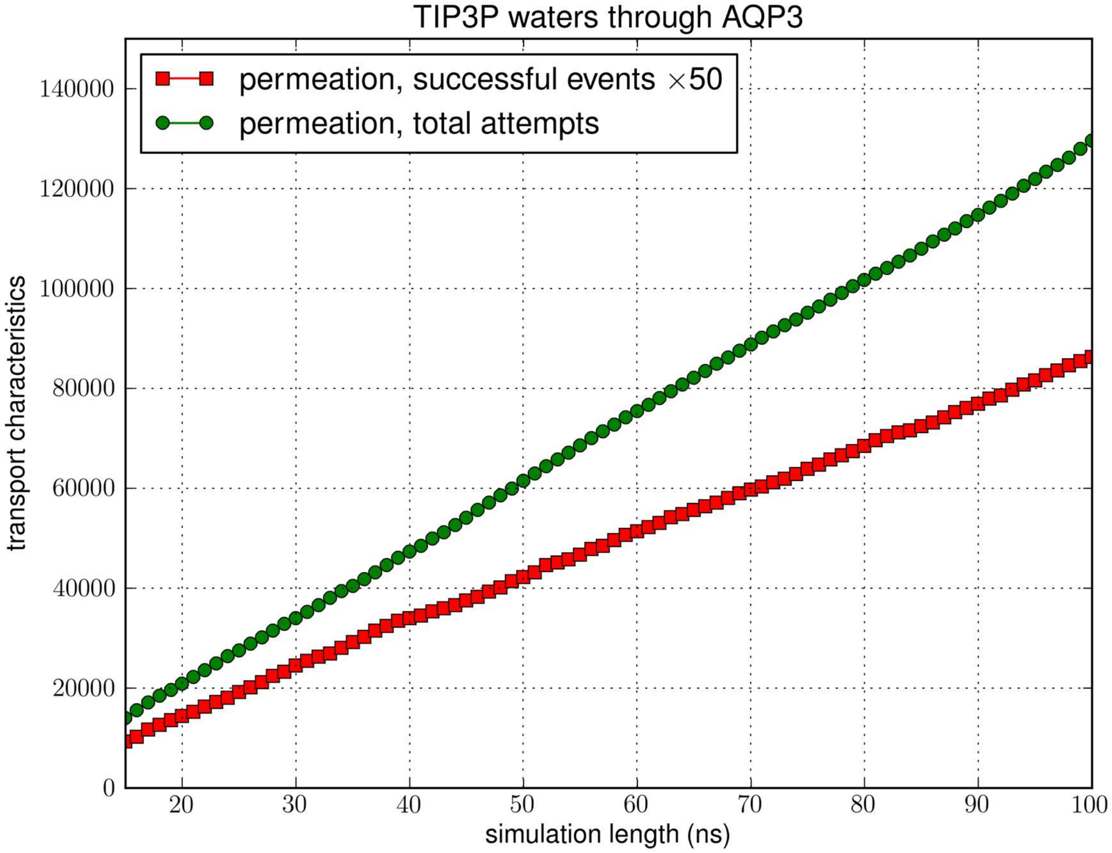


**Fig. S3a**. Transport characteristics of erythrocyte AQP3: the time course of the number of transport events *vs.* the number of attempts.

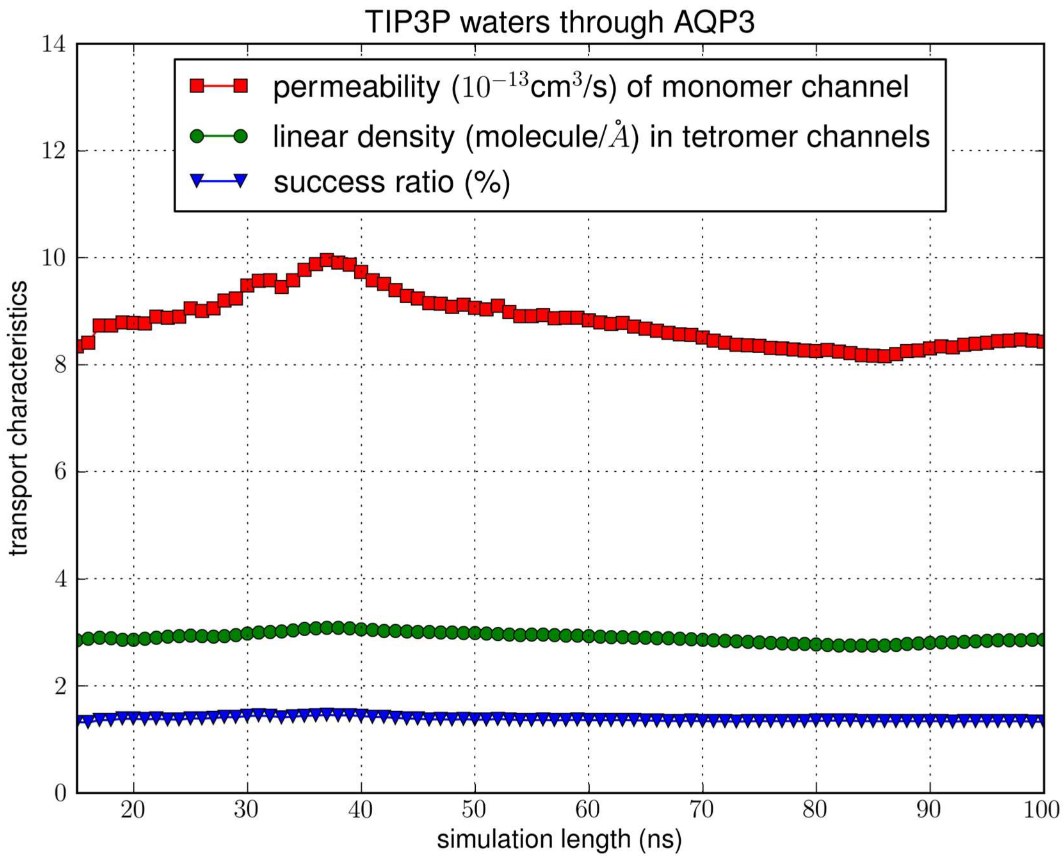


**Fig. S3b**. Transport characteristics of erythrocyte AQP3: convergence of the computation: the computed values of permeability, linear density, and success ratio *vs.* length of simulation.

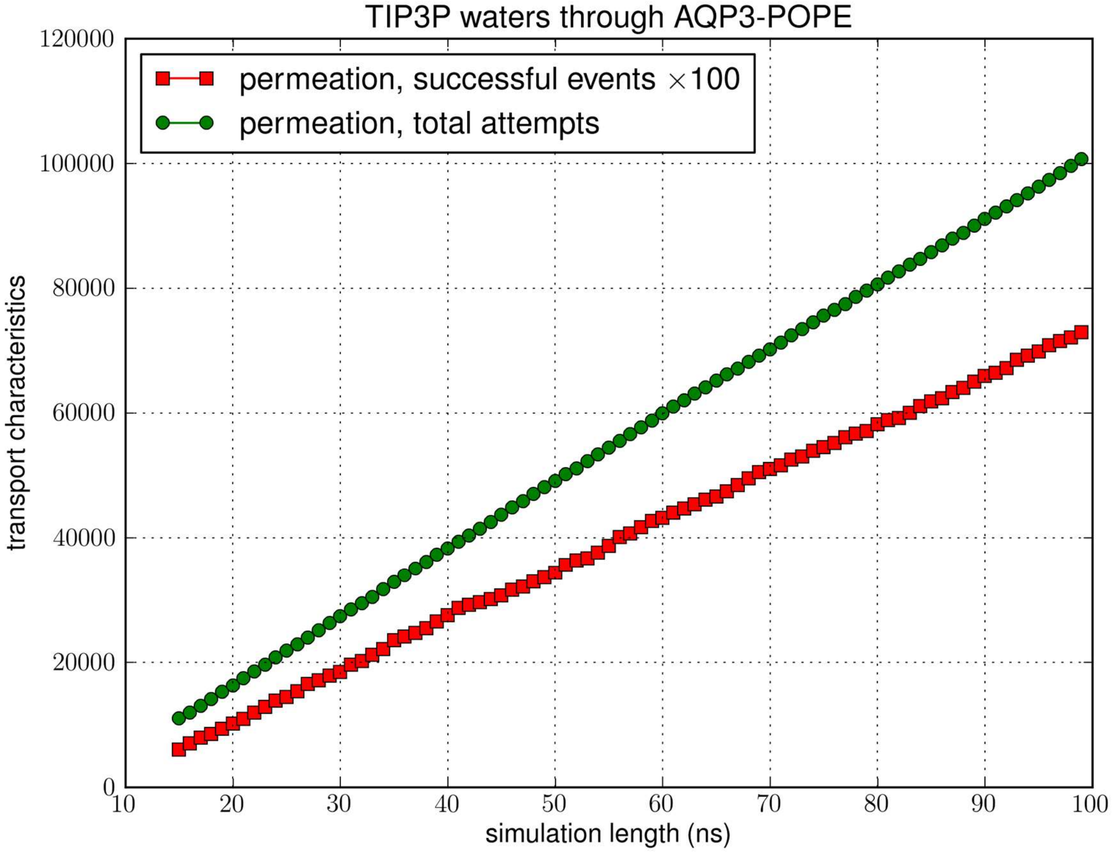


**Fig. S3c**. Transport characteristics of AQP3 embedded in POPE bilayer: the time course of the number of transport events *vs.* the number of attempts.

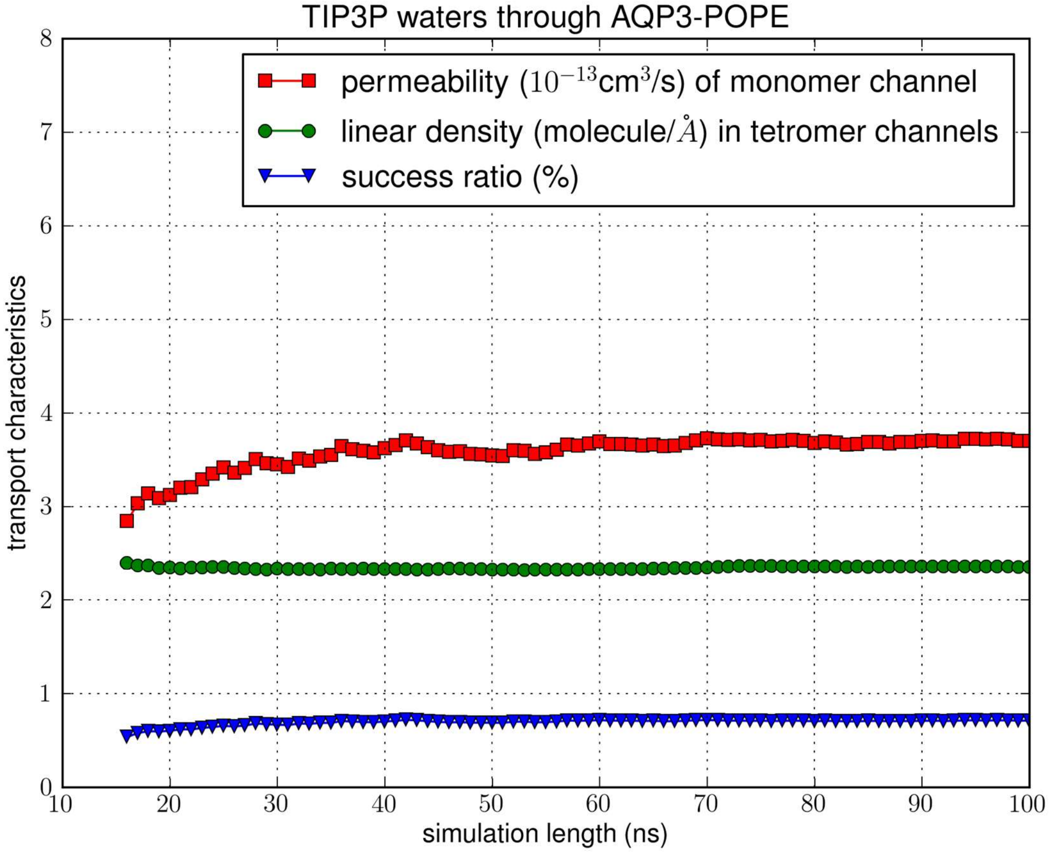


**Fig. S3d**. Transport characteristics of AQP3 embedded in POPE bilayer: convergence of the computation: the computed values of permeability, linear density, and success ratio *vs.* length of simulation.

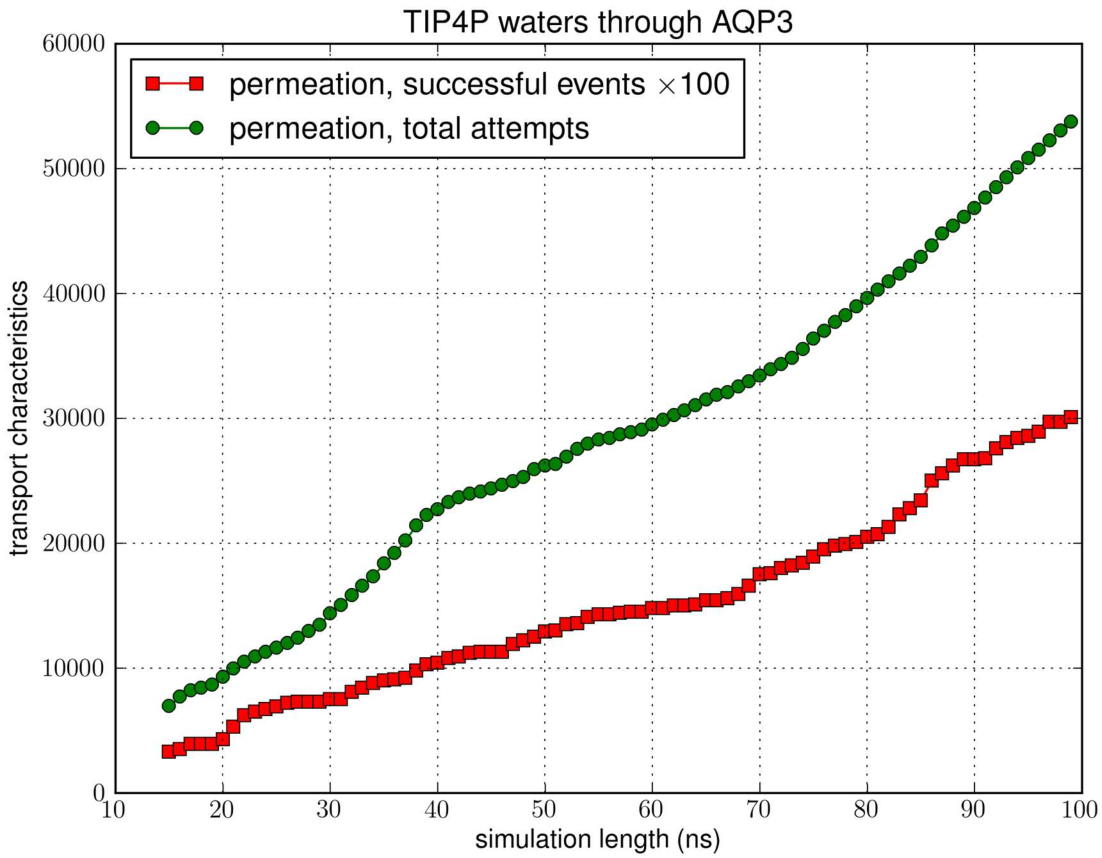


**Fig. S3e**. Transport characteristics of erythrocyte AQP3 with TIP4P water model: the time course of the number of transport events *vs.* the number of attempts.

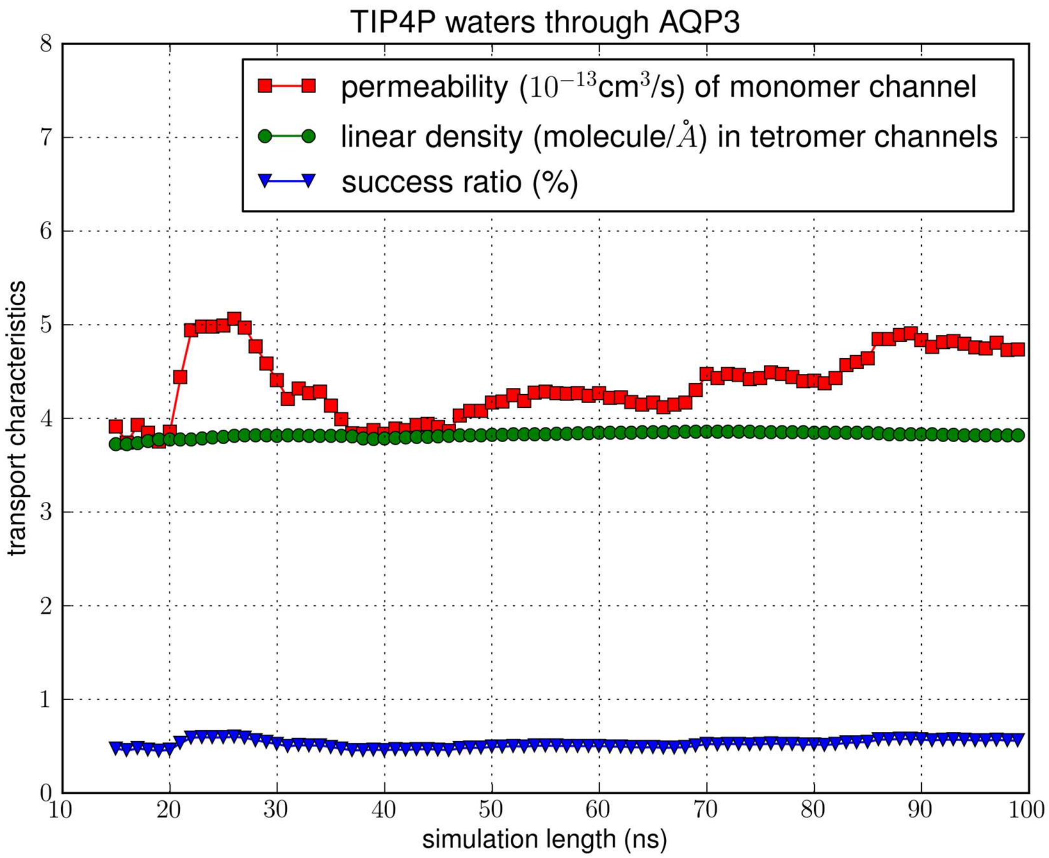


**Fig. S3f**. Transport characteristics of erythrocyte AQP3 with TIP4P water model: convergence of the computation: the computed values of permeability, linear density, and success ratio *vs.* length of simulation.

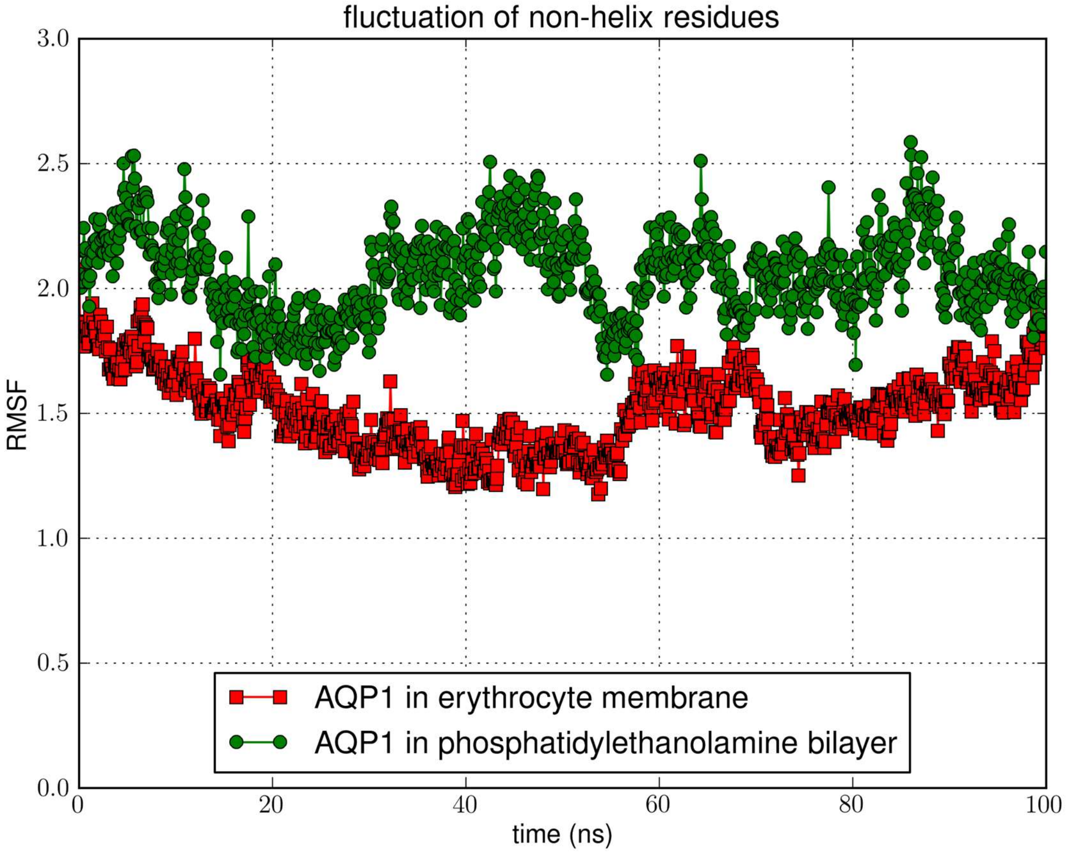


**Fig. S4**. Fluctuations of the non-helix residues of AQP1 in erythrocyte membrane *vs* in POPE bilayer.

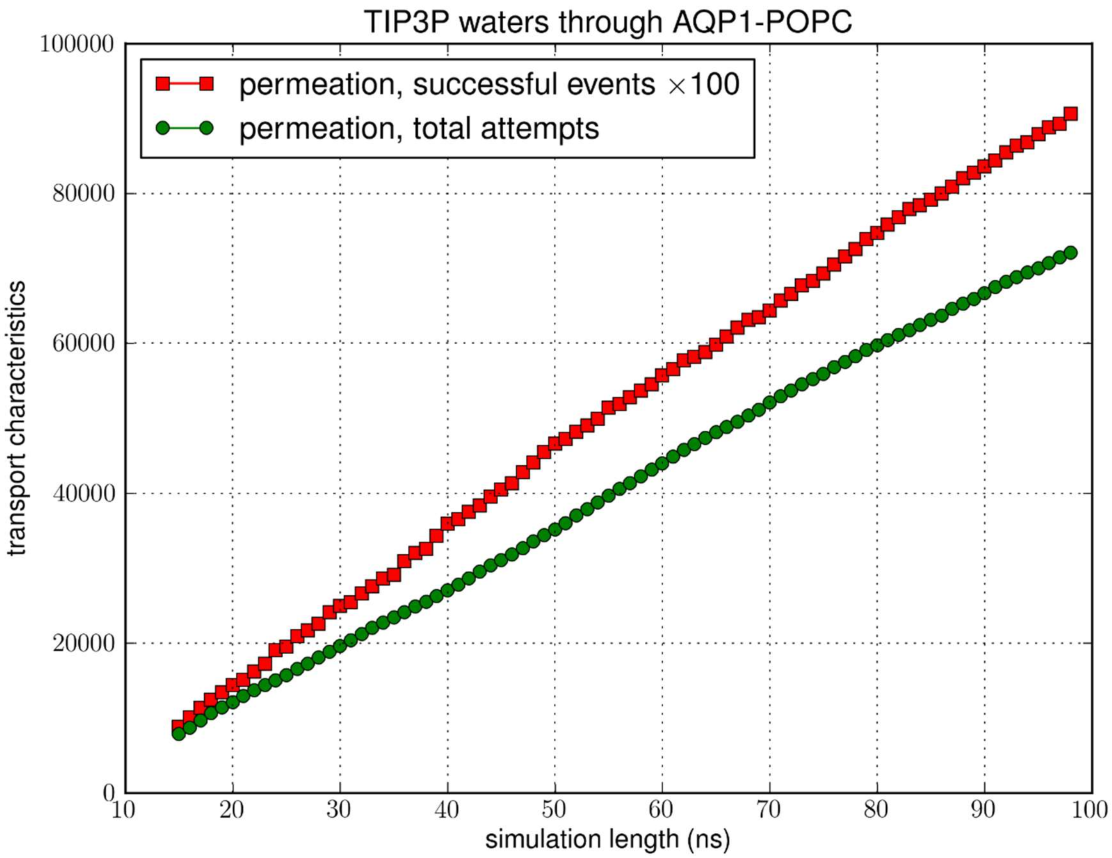


**Fig. S5a**. Transport characteristics of AQP1 embedded in POPC bilayer: the time course of the number of transport events *vs.* the number of attempts.

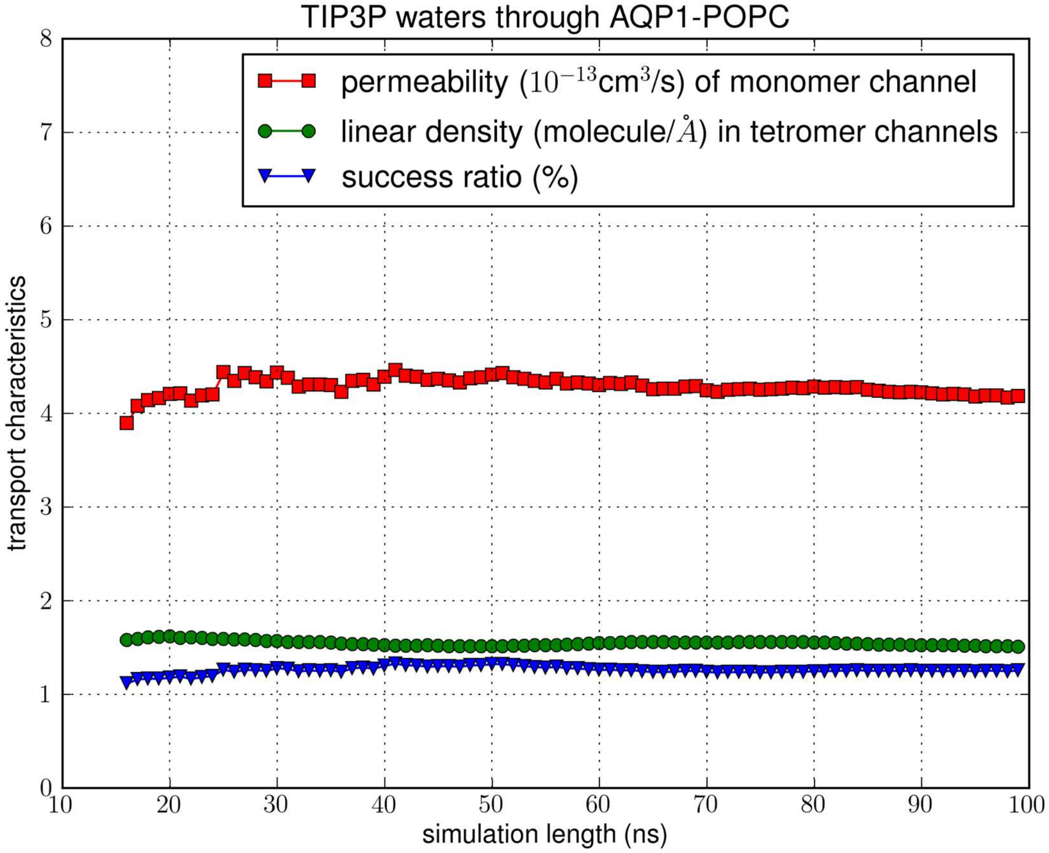


**Fig. S5b**. Transport characteristics of AQP1 embedded in POPC bilayer: convergence of the computation: the computed values of permeability, linear density, and success ratio *vs.* length of simulation.
